# Workplace bullying in midwifery: insights from a survey of New Zealand midwives

**DOI:** 10.1108/JHOM-05-2025-0272

**Published:** 2025-10-01

**Authors:** Vasudha Rao, Beth Tootell

**Affiliations:** Massey University – Manawatu Campus, Palmerston North, New Zealand

**Keywords:** Bullying in healthcare, Bullying New Zealand, Midwife bullying, Midwifery bullying, Student midwife bullying

## Abstract

**Purpose:**

The purpose of this study is to explore the prevalence and lived experiences of bullying among New Zealand midwives.

**Design/methodology/approach:**

A two-phase study was designed to investigate midwife bullying in New Zealand. Phase I, reported in this article, concentrated on objective measures of bullying and identifying areas of focus for Phase II, a qualitative interview-based study. The Negative Acts Questionnaire-Revised (NAQ-R; Einarsen *et al.*, 2009) was utilized via the Qualtrics platform to assess workplace bullying behaviours. In addition, open ended questions were included for each of the NAQ-R survey questions to gather a deeper insight into the kinds of bullying experienced by New Zealand midwives, as well as to guide us for the next phase (Phase II) of our study.

**Findings:**

The findings of this study revealed that midwife bullying continues to be a pervasive issue in New Zealand. Quantitative data from NAQ-R scores indicated a large component of severe bullying (44.3%). Qualitative data highlighted the complexities of where bullying occurred, where it can come from and how it appears in New Zealand. Data showed that bullying of midwives originated from authority figures with themes of exclusion, false narratives, victim blaming and fear of speaking up. Impacts of bullying on victims included mental health concerns, employee turnover and effect of student learning; as well as negative effects on patient care.

**Originality/value:**

The findings of this study highlight the intricacies of midwife bullying in New Zealand. Data from the objective NAQ-R instrument reveal that midwife bullying continues to be a persistent issue in New Zealand. Qualitative findings, help in contextualising the broader phenomenon of bullying amongst midwives, offering clarity on the dynamics at play within a specific New Zealand organisational or sociocultural settings. Results show that to tackle bullying effectively, a multi-faceted approach is needed including policy, and leadership. Results also help emphasise the need for further research in this area in the New Zealand context.

## Introduction

Workplace bullying is a widespread and ongoing issue worldwide, that continues to be of concern to organizations, researchers and practitioners. While several definitions of workplace bullying exist, it is broadly characterized by harassing, offending, socially excluding or negatively affecting someone's work tasks. Workplace bullying has been a longstanding issue in healthcare, with its traditionally hierarchical structures and power imbalances. In midwifery, bullying has been documented since the 1990s (Capper *et al.*, 2021; Gillen *et al.*, 2009; Killingley, 2016; Hogan *et al.*, 2018). The consequences of workplace bullying are wide-ranging and have been shown to have an impact on organizational functioning, and the physical and mental health of those affected, and most critically, in healthcare settings, on patient care. In midwifery, these effects can be quite acute, as they could lead to harmful outcomes for mothers and babies. Despite identifying workplace bullying as a significant issue in midwifery, improvements in mitigating/addressing this issue have been minimal, indicating that there is a deeply ingrained culture of bullying within midwifery.

Further, although awareness is increasing worldwide, workplace bullying among midwives remains insufficiently explored in New Zealand scholarship. The hierarchical structure of healthcare systems, alongside gendered expectations, intersectional dynamics and weak accountability mechanisms, contributes to an environment where bullying can be ignored or accepted as normal. While there has been research on nurse bullying in New Zealand, there is a significant gap in the understanding of bullying in the midwifery context, that can shed light on the prevalence of bullying as well as the covariables of bullying in the midwifery context (Capper and Thorn, 2022; Hawkins *et al.*, 2021). Although limited, research in this area indicates that bullied midwives may quit their jobs (Capper and Thorn, 2022). With the existing staff shortages and overall resource constraints and pressure in midwifery in NZ may be plagued with similar issues in the future, if not already existing. This is a compelling argument for further research in this area. This paper reports Phase 1 of a two-phase study aiming to explore the prevalence, nature and effects of bullying in midwifery in New Zealand. This paper focusses on three main research objectives: (1) to assess the prevalence of workplace bullying among New Zealand midwives using the NAQ-R instrument; (2) to investigate the lived experiences of bullying through qualitative responses and (3) to highlight key implications for policy development, leadership practices and future research directions.

## Literature

Workplace bullying is a global concern with well-documented negative consequences, attracting sustained interest from organisations, researchers and practitioners (Berry *et al.*, 2012; Fink-Samnick, 2017; Franklin and Chadwick, 2013; Hoel and Einarsen, 2020; Sauer and McCoy, 2017; Shorey and Wong, 2021). While definitions vary, widely accepted characteristics include “harassing, offending, socially excluding someone or negatively affecting someone's work tasks” (Einarsen *et al.*, 2003, p. 15). Related terms such as harassment, incivility, horizontal or vertical violence (depending on power differentials) and relational aggression, are also used to describe bullying (Cowan, 2012; Dellasega, 2009).

Bullying behaviours range from covert to overt actions, including sarcasm, criticism, exclusion and ostracism (Alexander *et al.*, 2021; Fenwick *et al.*, 2012; Javanmard *et al.*, 2020; Lang *et al.*, 2022; Sheehy *et al.*, 2021). Power abuse, racism and discrimination frequently underpin these behaviours (Fenwick *et al.*, 2012; Javanmard *et al.*, 2020). For example, international midwives may also face bullying linked to accents and foreign qualifications (Javanmard *et al.*, 2020).

### Bullying in healthcare settings

Workplace bullying can cause physical and psychological issues like anxiety, depression and even increase the risk of death (Verkuil *et al*., 2015). Healthcare workers are especially vulnerable to bullying (Liu *et al.*, 2019). It may hinder their ability to provide care and referrals, leading to high stress levels with serious consequences for themselves, patients and organizations (Lever *et al*., 2019; Purpora *et al*., 2019).

### Bullying in midwifery

While nursing has been widely studied, midwifery has received comparatively little attention (Hawkins *et al.*, 2021). Nonetheless, bullying persists within midwifery culture globally (Capper *et al.*, 2021; Capper and Thorn, 2022; Catling *et al.*, 2017), with research in Australia and the UK highlighting its prevalence over the past 3 decades (Capper and Thorn, 2022; Royal College of Midwives, 2022; Killingley, 2016).

### Organisational power structures

Hierarchies and power differentials within healthcare organisations create conditions conducive to bullying (Van Fleet and Van Fleet, 2022). Vertical bullying, where senior staff target juniors, is common among clinical midwives and students, particularly in clinical environments, often in the presence of mothers and newborns (Capper and Thorn, 2022). Mentors, managers and even doctors have been reported as perpetrators (Capper *et al.*, 2020; Catling and Rossiter, 2020; Harvie *et al.*, 2019; Javanmard *et al.*, 2020). Australian and New Zealand studies reveal that bullying often involves senior midwives targeting juniors (Capper and Thorn, 2022). These findings emphasise the impact of hierarchical dynamics on midwifery bullying (Catling and Rossiter, 2020; Fenwick *et al.*, 2012; Javanmard *et al.*, 2020).

New graduates and students are particularly vulnerable, as they often lack experience and occupy low-status positions (Catling *et al.*, 2017; Catling and Rossiter, 2020; Sheehy *et al.*, 2021). Midwifery students have been a particular focus internationally (Capper *et al.*, 2020; Capper and Thorn, 2022; Gillen *et al.*, 2009; Hegney *et al.*, 2006), given their susceptibility due to power imbalances (Lash *et al.*, 2006). In the UK, bullying has contributed to midwifery students prematurely exiting the profession (Royal College of Midwives, 2016). Despite long-standing awareness, improvements have been limited, pointing to a persistent bullying culture (Capper *et al.*, 2021; Hills *et al.*, 2018; Farr-Wharton *et al.*, 2023).

### Consequences

As in other health professions, bullying in midwifery has significant personal, organisational and clinical repercussions. These include psychological distress, absenteeism, burnout and turnover at the individual level, and workforce shortages at systemic levels (Capper *et al.*, 2021; Catling *et al.*, 2017; Hassard *et al.*, 2018; Hegney *et al.*, 2006; McKenna and Boyle, 2016; Yoshida and Sandall, 2013). In midwifery, bullying can also adversely affect mothers and infants. A recent UK report linked bullying, fear of reporting and cultural tolerance to the deaths of nine mothers and over 200 infants (Ockenden, 2022).

### Interventions

Addressing bullying requires both organisational and multi-level interventions (Gillen *et al.*, 2017). Strategies to gain mention include policy reforms, stress management and training on recognising harmful behaviours. However, existing policies often fail due to mistrust and fear of retaliation (Dixon-Woods *et al*., 2022). Fear of reporting leads to underreporting (Capper and Thorn, 2022). Recent research recommends anonymous surveys, trauma-informed support, safe reporting mechanisms and fostering respectful workplace cultures to address bullying effectively (American Medical Association, 2021; Darling *et al.*, 2023). Some advocate for targeted interventions to support individual midwives subjected to bullying (Farrell, 2007).

### The New Zealand context

In New Zealand, though there is some evidence of midwives experiencing bullying, data on its prevalence and characteristics are lacking. One Australian study, a context close to New Zealand, reports that 43% of midwives experienced bullying (Harvie *et al.*, 2019).

While global evidence exists, New Zealand-specific research on bullying of midwives remains limited. Given the role of local, national and cultural factors in shaping bullying (Salin, 2021; Smith and Robinson, 2019; Zheng *et al.*, 2024), further research is needed to explore the prevalence and lived experiences of bullying among New Zealand midwives. This is especially significant given that midwifery is currently estimated to be understaffed by 40% in New Zealand (Ewertowska and Staniland, 2024).

## Method

Given the need for a better understanding of midwife bullying in New Zealand a two-phase study was designed. Phase I, reported in this article, concentrated on objective measures of bullying and identifying areas of focus for Phase II, a qualitative interview-based study. Accordingly, in this Phase I study, the Negative Acts Questionnaire-Revised (NAQ-R; Einarsen *et al.*, 2009) was utilized to assess workplace bullying behaviors. The NAQ-R instrument is particularly valuable in gaining an insight into the variables associated with bullying and hence the ideal choice for our study (Einarsen *et al.*, 2009).

The NAQ-R questionnaire, developed by Einarsen *et al.* (2009), is a 22-item survey measuring exposure to work- and person-related negative behaviours over the previous six months. This 22-item instrument, which evaluates three bullying dimensions (work-related, person-related and physically intimidating behaviors), captures participant responses across five frequency categories: never, now and then, monthly, weekly and daily. Responses are scored from 1 to 5, allowing a maximum total score of 110. For this study, a score of 33 or higher were considered to indicate those in the preliminary stages of bullying (Notelaers and Einarsen, 2013), while a score of 45 or higher indicated severe workplace bullying. Einarsen *et al.* reported that the NAQ-R instrument demonstrated strong reliability, with a Cronbach's alpha of 0.90 for its 22 items. To further insight into the responses and a deeper understanding of the kinds of bullying experienced by New Zealand midwives, as well as to guide us for the next phase (Phase II) of our study, we also included open-ended questions at the end of each of the NAQ-R questions in the survey, constituting the qualitative aspect of this research.

### Participants and recruitment

Surveys were anonymous and recruitment was through advertisements placed in relevant midwifery journals, magazines, groups and social media to reach a wide cohort of midwives. The sample comprised registered and student midwives who experiencing bullying within the past five years. Survey participation was voluntary, with informed consent obtained through the Qualtrics platform. Participants were recruited over a 6-week period using a combination of purposive and snowball sampling. Approximate response rate could not be determined due to the open nature of recruitment. The final sample included 113 completed responses.

### Quantitative data analysis

Quantitative data were derived from the 22-item *Negative Acts Questionnaire-Revised (NAQ-R)* to measure exposure to workplace bullying behaviours. Respondents were asked to indicate the frequency with which they experienced each negative act over the past six months using a five-point Likert scale with the following response categories: *never*, *now and then*, *monthly*, *weekly* and *daily*. This scale enabled a nuanced understanding of the frequency and intensity of bullying-related behaviours in the workplace.

Data was analysed using *IBM SPSS* (version 29). Initial analysis involved descriptive statistics to determine the distribution of responses for each item. Percentages were calculated to identify the proportion of responses that fell within each of the NAQ-R's standard scoring bands, as established in the literature (Einarsen *et al*., 2009), which categorises levels of exposure to bullying, ranging from non-exposure to severe bullying. This categorisation helps to quantify not only the presence but also the intensity of bullying experienced by participants.

Subsequently, the analysis focused on the *thematic clusters* of bullying behaviours as outlined in Einarsen *et al*.’s (2009) NAQ-R framework. These include work related bullying (e.g. being withheld information, excessive monitoring); person related bullying (e.g. being humiliated, ignored or ridiculed) and physically intimidating bullying (e.g. threats of violence or actual physical intimidation). Frequencies were computed for each cluster to examine the prevalence and relative weight of different forms of bullying experienced by midwives. This cluster-level analysis facilitated the identification of distinct behavioural patterns and categories of bullying that were more frequently reported, thereby offering a clearer understanding of the workplace dynamics impacting midwives. The use of NAQ-R's validated scoring structure, combined with the cluster-based approach, ensured the robustness and interpretability of the findings related to workplace bullying exposure.

### Qualitative data analysis

The data were analysed using thematic analysis, as defined by Braun and Clarke (2006), which involves identifying, analysing and reporting patterns (themes) within the data. This flexible approach supported the study's aim of understanding respondents' experiences of bullying in their context. It allowed for both the organisation of data in rich detail and the identification of repeated patterns of meaning. Therefore, we could identify codes as well as explore the contextual meaning behind the codes (Joffe and Yardley, 2004). Coding was inductive and conducted using NVivo software. Two researchers independently coded the data using NVivo, and initial coding was inductive. Meaningful segments were coded without discarding any data, guided by existing theory but open to new insights. Codes were grouped into themes and subthemes, which were reviewed and refined for clarity and coherence. A thematic map was developed to explore relationships between themes, with adjustments made to ensure alignment with the research focus.

We sought to ensure rigour in this research by offering detailed explanations of methodological alignment, outlining the participant selection process and incorporating interview quotations to support the analysis (Baxter and Eyles, 1997). The research team met regularly to discuss theme development and ensure rigour through reflexive dialogue. The authors also maintained reflexive journals to acknowledge potential researcher bias and increase transparency. While no formal triangulation was undertaken, researchers reviewed selected transcripts to enhance trustworthiness. The authors also maintained reflexive journals to acknowledge potential researcher bias and increase transparency.

## Results

### Quantitative data

A majority of the respondents identified themselves as female (98.5%), were between 45 and 64 years old and New Zealand Europeans (72.1%). Most were New Zealand citizens (83.6%) and worked in community-based (59.4%) and hospital-based settings (56.5%).

#### NAQ-R/behaviour survey

Notelaers and Einarsen (2013) established two gold standards for identifying workplace bullying: a lower standard for those in the early stages of bullying and a higher standard for severe cases. The NAQ-R cut-off scores were determined using sensitivity and specificity measures. A score of 33–44 suggested preliminary bullying, and scores of 45 or above signified severe bullying. For this study, as outlined above a score of *33 or higher on NAQ-R* indicated *occasional bullying or in the preliminary stages of bullying*, while a score of *45 or higher* indicated *workplace bullying or targets of severe bullying* Notelaers and Einarsen (2013).

This study analysed a total of 113 completed surveys. Results (Table 1) showed that a total of 60.7% respondents were either occasionally bullied, or targets of severe bullying. Of those, 16.4% % of respondents scored between 33 and 44, and were thus classified as preliminary stages of bullying or occasionally bullied; while 44.3% scored higher than 45 and thus classified as workplace bullying or targets of severe bullying Notelaers and Einarsen (2013).

**Table 1:** Frequency of bullying

Standard of bullying	NAQ-R score	Frequency (%)
Occasionally bullied (preliminary stages of bullying)	NAQ-R score 33–44	16.4
Workplace bullying (targets of severe bullying)	NAQ-R score higher than 45	44.3

**Source(s):** Figure by authors based on Notelaers and Einarsen's (2013) classification

#### Latent cluster analysis

Einarsen *et al.* (2009) employed a latent class cluster approach to evaluate participant responses to the 22 items of the NAQ-R, identifying seven distinct bullying profiles. For the purposes of this study, three of those clusters were applied to midwives: work-related bullying (items 1, 3, 14, 16, 18, 19 and 21), person-related bullying (items 2, 4, 5, 6, 7, 10, 11, 12, 13, 15, 17 and 20) and physically intimidating bullying (items 8, 9 and 22).

In the original study by Einarsen *et al*. (2009), the expected frequencies for these clusters were 10%, 10% and 3% respectively. This study markedly revealed (Table 2) that the observed frequencies for all three clusters exceeded those reported by Einarsen *et al.* (2009). Notably, within the physically intimidating bullying cluster, item 22 (“threats of violence”) received a response rate of zero. However, this finding contrasted with qualitative comments describing threatening and violent behaviour, and is discussed further and unpacked in the qualitative data analysis section.

**Table 2:** Ranking of NAQ-R items with highest scores

Rank	Bullying cluster	Item number	Description
1	Person-related bullying	Item 6	Being excluded or ignored
2	Work-related bullying	Item 14	Having opinions ignored
3	Person-related bullying	Item 5	Spreading gossip

**Source(s):** Figure by authors

Among the items assessed monthly or weekly, the highest reported frequency was for “being excluded or ignored” (item 6) within the person-related bullying cluster, followed by “having opinions ignored” (item 14) in the work-related bullying cluster and “spreading gossip” (item 5), also under person-related bullying.

### Qualitative data

Each item on the NAQ-R was followed by an open-ended prompt, inviting respondents to provide additional comments or clarification. Qualitative responses were analysed thematically (Braun and Clarke, 2006), using NVivo software, with the aim of identifying underlying patterns and emergent themes as outlined earlier. After the initial coding, themes were identified and grouped into three main categories. The resulting themes, all of which reflected negative experiences, offered deeper insight into the nature and context of workplace bullying among participants. These findings not only enriched understanding of the quantitative data but also highlighted areas warranting further investigation. The themes are presented in three categories: person-related experiences, organisational or work-related issues and experiences specific to student midwives. Table 3 displays each theme with corresponding example quotes from participants.

**Table 3:** Illustration of themes from qualitative analysis of data

Theme	Sub-theme	Example quote
Person-related	Fear and avoidance	“I don't share my opinions with her … because I know she will attack me.”
Organisation-related	Leadership failures	“Apologies were given both times, which reflected a lack of true leadership.”
Student-specific	Emotional harm	“The mental abuse I experienced during my studies … was the worst I have ever encountered.”

**Source(s):** Figure by authors

#### Personal related issues

*Targeting*: Respondents reported being targeted through labelling related to their race and/or ethnicity (e.g. *“As a tangata whenua midwife who advocates strongly, I am often* *labelled* *confrontational”)*; sexual orientation (e.g. *“Her staff were abusive because she is in a gay relationship”*) and age or experience (e.g. *“She surrounds herself with younger, more malleable people”*).

*Fear:* Fear was frequently cited by victims as a significant factor: *“I don't share my opinions with her, around her, or even within the profession anymore, because I know she will attack me if I do.”* This fear also led to avoidance behaviors, with one respondent stating, *“I am hesitant to make suggestions to midwives due to past negative experiences. I avoid certain colleagues who come across as unapproachable and volatile.”* Respondents also expressed fear about the consequences of speaking up for themselves. For student midwives, this included the risk of academic repercussions: *“We were blamed and faced the possibility of failing our practical midwifery papers.”*

*Negative interactions*: Respondents described negative workplace interactions, including being *“yelled at for several minutes in front of another CL,”* and experiencing distressing emotions such as shame and a sense of being *“inferior.”* Respondents reported feeling excluded, not only from their immediate work environment, but also from the broader midwifery community*: “I felt devalued and excluded from the midwifery community.”* Another concern was the spreading of what respondents described as false narratives: *“complete fabricated lies about me,”* which they felt were *“professionally undermining”* and damaging to their reputation.

*Victim blaming*: Victims of bullying were frequently blamed instead of supported, as evidenced by comments such as being *“belittled for not coping with workplace pressure*” or dismissed, simply for appearing stressed: *“It was suggested I was stressed, without anyone listening to the actual cause.”* So, those perceived as compliant were deliberately targeted: *“Difficult clients were assigned to the staff member who would comply and not make a fuss.”*

*Threatening behaviour*: In contrast to the NAQ-R survey that reported no physically intimidating behaviour, a comment suggested otherwise: *“I have been threatened, locked in a drug room, physically pushed”* emphasising the significance of qualitative studies to get a deeper insight into bullying than just quantitative surveys.

#### Organisational issues/work related

*Bullying from authority figures*: Participants reported experiencing bullying from individuals in senior positions, including their own managers and senior leadership: *“Bullying is coming from management”.* Bullying by doctors was also reported*: “Doctors often challenge you publicly on the care given or question you in front of their team.”* Midwives described having their voices dismissed or disrespected, especially in culturally significant contexts: *“At many management meetings where I am the Māori voice, the senior doctor yells, rolls his eyes, and storms out.”* Other midwives also described similar dismissive behaviors, such as *“eye rolling when I am talking or during my handovers.”* The public nature of the bullying extended to digital communication, with examples *like “passive-aggressive comments directed at the victim in emails sent to multiple recipients.”*

*Undermining of competence*: Respondents reported that their professional competence was not acknowledged by their employing organisation, with one noting*: “Competence is questioned and disregarded.”* This was evidenced in comments like*: “Not being considered or offered courses appropriate to my position.”* Others reported barriers to career development: *“I was not allowed to take on any portfolio or champion roles. My direct line manager has stripped me of every role I've pursued over the last eight years.”* One respondent recounted an experience in which reporting a patient related medical issue to their manager was met with hostility*: “Instead of discussing the issue with me and trying to find the cause, she shouted at me.”*

*Culture of bullying*: Respondents highlighted an organisational culture where bullying was normalised: *“Gossip and bullying are part of the culture.”* Respondents noted a lack of effort to address this, with one stating, *“They focus on the system and funding, never on culture which isn't expensive to change.”* The workplace environment was also described as *“a very male-dominated doctor culture,”* where “*strong women are definitely not supported,”* leading to gender-based discrimination - particularly significant given that the majority of midwives in New Zealand are women (Midwifery Council. 2023).

*Leadership deficiencies*: Respondents cited a lack of accessible leadership support, such as *“there is no midwifery leadership on the floor often,”* as well as inadequate demonstrations of leadership: *“Apologies were given both times, which reflected a lack of true leadership.”*

*Lack of accountability*: Further, a lack of accountability for perpetrators was reported, with one noting, *“The individual who does this to me is still employed and cannot be reprimanded as its too hard to deal with in the employed space.”* Despite raising concerns through formal channels, little to no action was taken*: “I have supported multiple colleagues to make reports via management and speak up, and no actions have been taken.”* In some cases, the behaviour was simply tolerated*: “The behavior is allowed.”* As a result, bullies were often seen as facing no consequences*: “The bully is on a power trip and doesn't change even when called out.”*

*Career impact:* Respondents highlighted the damaging professional consequences of bullying, with one stating, *“She created and shared a narrative that was untrue and damaging to my career.”* This led some to consider quitting their jobs*: “EAP, a lawyer, mediation lawyer, and my family have advised me to quit.”* Organisations thus faced high turnover rates: *“There's a continual turnover of people in that role.”* Another consequence was a lack of growth opportunities: *“Job growth was stunted and not supported.”*

*Patient care*: One of the serious consequences of bullying in healthcare is its impact on patient outcomes. As noted in the survey, it *“has affected both the midwife's ability to do their job and the woman's (patient) experience/outcome.”*

#### Student midwives

Student midwives reported specific challenges related to their emotions, progress and academic journey, including completing their studies and passing exams. One described the need for constructive feedback in performance reviews, but instead often facing *“blame, shame, and gossip.”* Mental abuse was also highlighted*: “The mental abuse I experienced during my studies and placement was the worst I have ever encountered.”* In some cases, situations arose that had potential legal implications: *“Legally, I knew students should not be left unattended with laboring women, especially during the last stage of labor.”* Exclusion also had a negative impact on their placements, which are crucial for their development as registered midwives: *“I did not feel supported during my studies or placements, nor was I given the same opportunities.”*

## Discussion

The aim of this study was to get an overview of midwife bullying in New Zealand and to gather data that would inform further exploration of bullying. The findings of this study highlighted the complexities of where bullying occurred, where it can come from and how it appears. The NAQ-R survey, an objective measure, was utilised along with open-ended questions to gain a more comprehensive view of workplace bullying of midwives. This approach revealed that midwife bullying continues to be a pervasive issue in New Zealand.

Of the midwives who self-identified as being bullied, more than half (60.7%) were objectively bullied. Of those, 44.3% were severely bullied and 16.4% were in the preliminary stages of bullying per NAQ-R survey data. This aligns with international literature indicating high prevalence rates of bullying in healthcare, including midwifery (Harvie *et al.*, 2019; Capper and Thorn, 2022) and conforms to Notelaers and Einarsen's (2013) classification criteria. However, while the survey results showed no physically intimidating behaviours, qualitative comments referenced threats and violent conduct. This discrepancy may suggest underreporting or limitations in how the NAQ-R captures physical intimidation which is a recognized aspect of bullying in midwifery (Gillen *et al.*, 2009; Newman *et al*., 2019).

Qualitative data revealed the nature of bullying, highlighting that midwives were mostly bullied by those in senior positions, and doctors. Some respondents reported being targeted due to ethnicity and/or sexual orientation. A lack of accountability for perpetrators was a recurring theme. These findings are consistent with global literature and underscore themes of exclusion, false narratives, victim blaming and fear of speaking up (Javanmard *et al.*, 2020; Capper and Thorn, 2022; Alexander *et al.*, 2021; Fenwick *et al.*, 2012).

Workplace bullying leads to lower job satisfaction and a higher likelihood of employees wanting to leave their job or even the profession altogether (Cakal *et al.*, 2021), which was also evident in the responses to this survey. Bullying is a significant source of stress for victims in the workplace (Lever *et al*., 2019; Purpora *et al*., 2019), and in the healthcare profession presents significant risks to patient care as well (Ockenden, 2022). Research involving nurses has shown a link between reported bullying and serious or potentially serious medical errors (Chan *et al.*, 2022). Our findings similarly highlighted the potential for inadequate care of patients, including unreported errors, reduced vigilance and delays in treatment. Changes in attitude caused by bullying can also undermine care quality, risking physical and emotional harm to patients (Atta *et al.*, 2025).

Participants commonly cited challenges such as having their professional abilities questioned and facing obstacles in career advancement, which is consistent with existing research on workplace bullying (Capper *et al.*, 2020; Catling *et al.*, 2017). Despite widespread reports of normalised bullying cultures in the literature, our study found a lack of effective responses to combat this culture, indicating a need for more proactive institutional measures. Power imbalances embedded within hierarchical organisational structures can foster environments that increase the likelihood of bullying (Einarsen *et al*., 2020). When authority and control are centralized at the top levels, employees lower in the hierarchy may feel powerless and hesitant to confront unfair or aggressive actions. This imbalance of power can result in leaders misusing their position, creating a workplace culture where bullying is ignored or accepted. The findings of this study allude to such an organizational culture in midwifery.

Applying organisational culture theory, bullying flourishes in environments characterised by weak accountability, normalised aggression and ambiguous leadership practices (Quinn *et al*., 2025). Furthermore, social learning theory (Bandura and Walters, 1977) provides insight into how senior staff behaviour shapes organisational norms, suggesting that when leaders tolerate or model bullying, such behaviours are likely to proliferate.

Organisationally, bullying often originated from authority figures, such as managers and senior leadership, a trend confirmed by other studies (Catling and Rossiter, 2020; Harvie *et al.*, 2019). According to the respondents, this was compounded by a lack of leadership support when bullying occurred. The Integrated Competing Values Framework (ICVF) highlights the need for leaders and managers to adapt their leadership behaviors based on context (Vilkinas and Carton, 2006). It follows that leaders/managers must possess a diverse set of skills and operational capabilities to effectively navigate the complex emotions, situations and challenges inherent in workplace bullying. When addressing complex issues such as workplace bullying, this requires adopting the role of an integrator which involves balancing competing values, fostering collaboration and adapting leadership approaches to navigate complex issues like workplace bullying (Vilkinas and Carton, 2006). The findings of this study suggest that, when placed in leadership or managerial roles, midwives did not adopt the “integrator” role outlined in the Integrated Competing Values Framework (ICVF) during bullying incidents, thereby limiting their effectiveness in managing such situations.

Employees tend to imitate the behaviors shown by their leaders; when leaders demonstrate respectful and inclusive actions, the likelihood of bullying decreases according to the social learning theory (Bandura and Walters, 1977). Conversely, when leaders tolerate, overlook or participate in aggressive or authoritarian behaviors, they can foster a culture where bullying becomes accepted. Therefore, leadership behaviors have a direct impact on the organisational climate, shaping it into either a supportive environment or a toxic one susceptible to bullying and the results of this study highlight the importance of focusing on leadership behaviour of midwives. Indeed, it has been found that leadership styles that emphasize relationships, core values, ethical behavior and adaptability are strongly associated with lower levels of workplace aggression and bullying, while passive or abusive leadership tends to contribute to higher rates of such harmful behaviors (Cao *et al*., 2023).

The experiences of student midwives presented unique challenges. Themes of blame, shame and mental abuse closely mirrored the literature (Hegney *et al.*, 2006; Gillen *et al.*, 2009), though our study raised additional concerns about the legal implications of bullying which is less explored in existing research. Furthermore, while past studies note a lack of support during placements, this study provides detailed narratives illustrating how exclusion and negative interactions impact student learning and well-being.

The concept of intersectionality is especially relevant in the New Zealand context, and viewed through an intersectional lens, the findings of this study highlight how the overlapping identities of ethnicity, gender and age can heighten the risk of bullying for Māori and LGBTQ + midwives. Intersectionality examines how overlapping identities, such as gender, ethnicity and age, shape distinct and often more marginalized experiences, which are frequently missed in research that considers these identities in isolation (Christensen and Jensen, 2012). Thus, intersectionality helps explain how multiple forms of discrimination can intersect to produce unequal experiences (Cho *et al*., 2013). Particularly specific to the New Zealand context was the targeting of Māori midwives, helping to fill a gap in literature where localized data has been scarce.

Overall, the findings underscore the importance of understanding bullying not simply as interpersonal conflict, but as a manifestation of deeper structural inequities. The interplay of professional hierarchies, cultural marginalisation and weak leadership creates conditions that perpetuate harm. The results highlight that workplace bullying is not merely a collection of personal disputes, but a reflection of deeper, systemic and organisational imbalances. Conceptually, according to organisational culture theory, bullying flourishes in environments characterised by weak accountability, normalised aggression and ambiguous leadership practices (Quinn *et al*., 2025). Furthermore, social learning theory (Bandura and Walters, 1977) provides insight into how senior staff behaviour shapes organisational norms, suggesting that when leaders tolerate or model bullying, such behaviours are likely to proliferate. Further, findings also emphasise the complex role of leadership in addressing bullying (Woodrow and Guest, 2017). They indicate the need for culturally sensitive and change oriented leadership is needed to combat bullying in midwifery (Cao *et al*., 2023; Hutchinson and Hurley, 2013). This is especially relevant in hierarchical professions such as midwifery, where authority figures serve as role models and gatekeepers to career progression.

## Conclusions/implications

This study offers a critical insight into the prevalence and complexity of workplace bullying among midwives in Aotearoa New Zealand. The use of a validated quantitative tool (NAQ-R) alongside rich qualitative data highlights the layered and often intersecting nature of bullying experiences, particularly for vulnerable groups such as Māori and LGBTQ + midwives and midwifery students. Bullying emerges not merely as interpersonal conflict, but as a manifestation of systemic issues linked to leadership practices, organisational culture and entrenched power structures. Addressing this issue requires a comprehensive strategy that incorporates culturally responsive leadership development, confidential reporting channels and robust accountability frameworks. The findings contribute to both theory building, by illuminating how bullying is structured and sustained, and to practice, by identifying actionable steps healthcare organisations can take to mitigate harm.

The findings underscore that bullying within midwifery is a serious issue requiring a comprehensive response, including targeted policy measures and effective leadership. Participant responses offer important insights into recurring patterns, tensions and lived experiences that can inform and refine theoretical understanding. The qualitative data, in particular, brings to light subtle themes and complexities often missed by quantitative tools, supporting theory development by revealing new conceptual links and questioning established assumptions. By situating individual experiences within broader frameworks of workplace culture and structural power, the study demonstrates how bullying functions both as a personal encounter and a systemic organisational issue. These insights are especially valuable in contextualising the phenomenon within New Zealand, shedding light on the specific organisational and sociocultural dynamics affecting midwives.

From a practice and policy standpoint, the findings suggest that healthcare institutions should implement culturally safe leadership training and establish confidential reporting mechanisms. Policies must account for intersectional vulnerabilities and ensure meaningful accountability among senior staff. Incorporating bullying prevention into mentorship programs could provide better support for students and early career professionals. Institutions must also respond to reports promptly and fairly, reinforcing a culture of trust and safety.

Leadership in healthcare and midwifery has a critical role in fostering inclusive, supportive environments, particularly for those most at risk. This includes taking proactive steps to dismantle entrenched bullying cultures, especially in view of respondents' concerns about institutional inaction and the persistent normalization of bullying within the midwifery profession, as highlighted in existing literature. Further, previous studies have found that proactive advocacy driven relationships such as sponsorship, where a senior leader (sponsor) actively supports the career progress of a sponsee, can play a role on mitigating bullying in the context of nursing (Rao and Tootell, 2024). This involved protection from consequences of bullying, reporting and ensuring a positive outcome for the victims of bullying. This is something to consider in the similarly hierarchy driven context of midwifery.

## Limitations and future research

The participation criteria of this study may have excluded those who subjectively may not perceive themselves as being bullied, that could have been objectively clarified with the NAQ-R survey. Additionally, some midwives may have chosen not to participate out of fear of retribution, given the stigma surrounding the topic and the nature of the profession especially in a small nation like New Zealand. The study's demographics may also be skewed, due to the small sample size.

While the number of responses to the survey's open-ended and quantitative components is relatively small, their contribution to this research should not be underestimated. In early-stage or exploratory studies, especially those investigating complex or under-researched phenomena, smaller datasets can offer disproportionately rich insights (Denzin and Lincoln, 2018). As with many mixed-methods studies, qualitative interpretation is subject to researcher bias, though steps were taken to enhance rigour. Rather than aiming for generalisability at this stage, the study is designed to map the terrain and guide the development of more targeted instruments for subsequent quantitative phases or in-depth qualitative interviews. As such, even a modest set of well contextualised responses can serve as a powerful foundation for iterative research that combines conceptual development with empirical rigour.

Given the findings of this research, further research is warranted to investigate the underlying causes of bullying within midwifery and to explore individual experiences through in-depth qualitative inquiry. Such work is essential to generating richer, more nuanced understandings of the issue, which can inform the development of sustainable and effective interventions. Future studies should examine the structural and organisational factors that perpetuate bullying, employing frameworks from organisational behaviour to assess the impact of targeted strategies. Future, longitudinal research is needed to delve deeper into structural and leadership factors and evaluate the long-term effectiveness of bullying prevention interventions. Phase II of this study will involve semi-structured interviews to further explore the lived experiences of midwives in the New Zealand context, contributing to a deeper understanding of how bullying operates within specific sociocultural and institutional settings.
